# Machine learning reveals immune heterogeneity in recovery-phase samples of pediatric wheezing

**DOI:** 10.3389/fped.2026.1878743

**Published:** 2026-08-24

**Authors:** Jianhua Ben, Lingyan Wu, Luyun Tong, Yi Jin

**Affiliations:** 1 The People's Hospital of Longyou County, Clinical Lab, Quzhou, China; 2 The People's Hospital of Longyou County, Department of Pediatrics, Quzhou, China; 3 Sir Run Run Shaw Hospital, Clinical Lab, Hangzhou, China

**Keywords:** biomarker, early identification, immune infiltration, machine learning, wheezing

## Abstract

**Background:**

Pediatric wheezing diseases are linked to the risk of progression to persistent wheezing or asthma, particularly in cases with chronic airway inflammation and genetic predisposition. Early identification of high-risk children is crucial for timely intervention, yet the recovery phase of wheezing episodes, a transitional period, is often overlooked.

**Methods:**

We integrated RNA-seq data from GEO datasets (GSE113209 and GSE103166), focusing on 39 recovery-phase nasal mucosal samples from two independent cohorts. After log-transformation and batch-effect correction using ComBat, differential expression analysis was performed using the limma-trend method. Hierarchical clustering identified two distinct clusters, and differential gene expression (DEGs) was analyzed using GO, KEGG, GSEA, and DO methods. Immune infiltration patterns were assessed with CIBERSORT. Machine learning approaches (lasso regression, SVM-RFE) identified key biomarkers for potential subgroups in recovery samples.

**Results:**

Clustering revealed two clusters with differential immune infiltration patterns. Cluster 2 exhibited increased eosinophil, mast cell, and memory B cell infiltration. DEGs included 791 upregulated and 517 downregulated genes (|log2FC| >0.25, *p* < 0.05; 1,273 genes remained significant at FDR <0.05), highlighting immune activation pathways. Machine learning identified FLNA, PTGDR2, RNASE3, and IL18R1 as key biomarkers, predictive of cluster differentiation. In an external cohort, IL18R1 predicted recurrent wheezing in children with the first onset.

**Conclusions:**

Recovery-phase samples of wheezing illness exhibit heterogeneity. Cluster analysis revealed a subset demonstrating propensity for pro-inflammation, while biomarkers identified through machine learning correlated with immune infiltration. Furthermore, using external cohort, we confirmed the association between IL18R1 expression in recovery-phase samples and recurrent wheezing episodes. The pro-inflammatory subpopulation identified in recovery-phase samples warrants further investigation.

## 1 Introduction

Wheezing diseases in infants and children are closely linked with an increased risk of developing persistent wheezing and asthma later in life (1, 2). Wheezing in early childhood can arise due to viral infections, environmental exposures, or genetic predisposition (3–5) While many children outgrow these symptoms as they age, a subset of pediatric patients will experience a progression from transient wheezing to chronic, persistent wheezing (6, 7). Although early screening for childhood asthma is crucial for disease management, implementing comprehensive monitoring for all wheezing children proves to be a resource-intensive practice with limited clinical returns (2, 8, 9). This necessitates the development of more precise risk stratification approaches derived from clinical practice to identify high-risk populations warranting targeted early intervention.

**Table 1:** Primer design.

Primer	Sequence
GAPDH	Forward 5’- GGAGCGAGATCCCTCCAAAAT-3’
	Reverse 5’- GGCTGTTGTCATACTTCTCATGG-3’
IL-18R	Forward 5’- AAGAACGCCGAGTTTGAAGAT-3’
	Reverse 5’- GAGCAGTTGAGCCTTACGTTT-3’

Bronchiolitis, often caused by respiratory syncytial virus (RSV) or rhinovirus, is a common wheezing-related condition in infants and can be an initial trigger for recurrent wheezing episodes (10). Studies have highlighted that children with severe bronchiolitis, especially those with genetic or allergic predispositions, are at a significantly higher risk for developing asthma (3, 5). The frequent association between early-life viral-induced wheezing and asthma emphasizes the importance of timely diagnosis and intervention, as prolonged airway inflammation may lead to structural changes in the respiratory tract, contributing to chronic symptoms and exacerbations (5, 11, 12).

Given this potential progression, we propose that during the recovery phase of bronchiolitis, it is essential to identify children at high risk of recurrent cough and wheezing episodes. Although not all these children will eventually develop asthma, this stratified approach would significantly refine the target population while minimizing oversight of at-risk individuals (13).

Nevertheless, the heterogeneity in infection profiles among pediatric patients, combined with the interplay of genetic and environmental factors, renders current screening tools inadequate for personalized risk assessment. Emerging methodologies, such as molecular profiling and immune signature analysis, hold promise for enhancing early identification and precision management of pediatric respiratory diseases. With high-throughput RNA sequencing (RNA-seq) and immune profiling techniques, we can begin to identify specific molecular markers and immune cell infiltration patterns associated with different disease trajectories (14–17) Immune cell composition, for instance, has been found to correlate with overall respiratory health outcomes, with increased infiltration of cells like eosinophils and mast cells associated with a higher risk of allergic diseases. Early detection of these markers during the recovery phase of a wheezing episode may allow for timely intervention and potentially mitigate the progression to asthma (10, 18, 19). However, current investigation of recovery-phase samples following initial wheezing episodes remains limited. Therefore, we utilized RNA-seq data from the Gene Expression Omnibus (GEO) database, specifically examining upper respiratory samples from pediatric patients during the recovery phase of wheezing illnesses. By integrating machine learning approaches, we investigated post-bronchiolitis heterogeneity in pediatric recovery-phase samples and explored potential biomarkers, subsequently validating associations between the identified markers and clinical outcomes in an independent validation cohort. This strategy aims to facilitate early biomarker-driven identification of children prone to recurrent wheezing episodes, thereby refining screening strategies for persistent wheezing risk while minimizing unnecessary surveillance.

## 2 Methods

### 2.1 Datasets

This study utilized datasets GSE113209 and GSE103166 from the Gene Expression Omnibus (GEO) database. The high-throughput RNA-seq data from these datasets were used in their TPM (Transcripts Per Million) form, as provided by GEO. A total of 39 recovery-phase samples were extracted from both datasets (20 from GSE113209, which included infants with viral bronchiolitis, and 19 from GSE103166, which included children recovering from asthma exacerbations).

To prepare the datasets for differential expression analysis, the transcript per million (TPM) values were first log-transformed using the formula log_2_(TPM+1). This step was necessary to stabilize the variance across highly expressed genes, as raw count matrices were not publicly available for all cohorts. Following the log-transformation, the integrated datasets underwent batch effect correction utilizing the ComBat function from the sva R package to minimize technical variations between the two independent studies. For the identification of differentially expressed genes, we employed the limma R package. Given that our input consisted of pre-normalized, log-transformed data rather than raw counts, we applied the standard empirical Bayes approach tailored for continuous expression data (via the limma-trend method), rather than the voom transformation. For the 39 recovery-phase samples, comprehensive demographic analyses, including age and gender, were conducted (Supplementary File S1). Notably, detailed demographic metadata for GSE103166 were unavailable directly through the GEO database and were manually curated from the corresponding publication (PMID: 30745463).

### 2.2 Clustering analysis

To identify distinct subgroups within the recovery-phase samples, we conducted hierarchical clustering analysis using the hclust function in R. This method employs a bottom-up clustering approach based on the Euclidean distance between samples, allowing for the exploration of inherent group structures within the dataset. The dendrogram was cut at a height determined by visual inspection of the clustering structure, yielding two distinct clusters. The cutting height was selected based on the largest gap between successive fusion levels in the dendrogram, as a data-driven approach to avoid imposing an arbitrary number of clusters.

### 2.3 Differential expression analysis (DEGs)

Differentially expressed genes (DEGs) between identified clusters were calculated using the limma R package, which applies linear modeling to estimate differences in gene expression across groups. Genes with |log2 fold change| >0.25 and a nominal *p*-value < 0.05 were considered significant DEGs (*n* = 1,308; 791 upregulated, 517 downregulated). Benjamini-Hochberg false discovery rate (FDR) correction was applied; 1,273 DEGs remained significant at FDR <0.05. Subsequent enrichment and machine learning analyses were based on the *p* < 0.05 DEG set.

### 2.4 Enrichment analysis

To investigate the functional significance of the DEGs, we performed various enrichment analyses using the ClusterProfiler R package: GO (Gene Ontology) Analysis: Enriched biological processes, molecular functions, and cellular components associated with DEGs were identified. KEGG (Kyoto Encyclopedia of Genes and Genomes) Pathway Analysis: We identified enriched signaling pathways among the DEGs to explore potential mechanisms. GSEA (Gene Set Enrichment Analysis): Conducted using the pre-ranked option, with genes ordered by the limma-moderated t-statistic, to examine enrichment of broader gene sets in pathways or processes. DO (Disease Ontology) Analysis: This analysis assessed the association between DEGs and various disease categories, including bronchial diseases and asthma.

### 2.4 Immune infiltration analysis

Immune cell composition within each sample was estimated using CIBERSORT with the LM22 reference signature matrix (1,000 permutations), a deconvolution algorithm that calculates the relative abundance of 22 immune cell types from bulk RNA-seq data. The output from CIBERSORT was used to analyze immune infiltration patterns across clusters, providing insight into potential immune mechanisms underlying disease progression.

### 2.5 Machine learning methods

To identify potential biomarkers predictive of persistent wheezing or asthma progression, we applied two machine learning methods in R: Lasso Regression: Using the glmnet R package, we performed lasso regression with 10-fold cross-validation to select key features from the DEGs. The optimal regularization parameter (lambda) was chosen based on the minimum cross-validation error (lambda.min). To evaluate predictive performance while accounting for the limited sample size (*n* = 39), leave-one-out cross-validation (LOOCV) was performed: in each of 39 iterations, logistic regression models were trained on 38 samples and tested on the single held-out sample. Pooled LOOCV predictions were used to compute cross-validated AUC with 95% confidence intervals via the DeLong method (pROC package). SVM-RFE (Support Vector Machine—Recursive Feature Elimination): Using the e1071 R package, we conducted SVM-RFE to iteratively remove the least significant features, refining the candidate biomarkers to those most indicative of progression risk. The overlapping features identified by both algorithms were retained as the most robust candidate biomarkers.

### 2.6 Acquisition of disease-related genes from geneCard

Asthma-related genes and persistent wheezing-associated genes were acquired from the GeneCards database. Genes were filtered according to their Relevance Score, yielding 1,082 candidate genes with relevance scores ranging from 1.85 to 22.75 (median = 2.98) (Supplementary File S2). Subsequently, differentially expressed genes (DEGs) were overlapped with the GeneCards-derived genes to explore potential associations with the disease.

### 2.7 Cohort data and sample collection for children with the first onset of wheezing

The external cohort was enrolled between January 2025 and December 2025 at the pediatric outpatient department and inpatient ward of The People's Hospital of Longyou. Inclusion criteria for the study: (1) Children and their guardians who provided informed consent to participate in the study; (2) Aged between 0 and 36 months; (3) Children presenting with initial upper respiratory tract symptoms; (4) Participants' guardians have no mental disorders, can communicate without barriers, and are able to independently complete the questionnaire. Exclusion criteria: (1) known immunodeficiency or chronic systemic disease; (2) use of inhaled or systemic corticosteroids within 14 days prior to enrollment; and (3) prior hospitalization for respiratory illness within the preceding 3 months. Basic demographic information was recorded, and viral infection status was assessed, including the detection of Influenza A (H1N1), Influenza B, Respiratory Syncytial Virus (RSV), Adenovirus, Rhinovirus, and Parainfluenza Virus, all of which are routinely tested at our hospital. Follow-up visits were required after symptom improvement. Nasopharyngeal swab samples were collected during the recovery phase (defined as 24–72 h after resolution of fever and acute respiratory distress, at the discretion of the attending clinician) to assess IL18R1 levels using quantitative PCR (GAPDH as housekeeping gene) (Table 1). Participants were monitored for reoccurrence of wheezing symptoms within 3 months via telephone interview and/or in-person follow-up visits, with recurrent wheezing determined by physician diagnosis.

A total of 110 pediatric participants were included in this study. During the 3-month follow-up period, 50 cases of positive outcomes and 52 cases of negative outcomes were recorded, with 8 participants lost to follow-up.

### 2.7 Statistical analysis

To analyze gene expression differences between the two clusters, Student's *t*-test was employed, comparing mean expression levels across samples to identify statistically significant differences. For assessing the classification performance of key genes, we calculated the Receiver Operating Characteristic (ROC) curve and Area Under the Curve (AUC) using the R package pROC. For differential expression analysis, Benjamini-Hochberg false discovery rate (FDR) correction was applied to adjust for multiple comparisons. All statistical analyses were conducted in R software (version 4.2.1). Leave-one-out cross-validation (LOOCV) was implemented using custom R scripts with the pROC package for AUC computation and DeLong 95% confidence intervals. 2^(-ΔΔCT) method was used for RT-qPCR result analysis. Statistical significance was denoted as follows: *p* < 0.05 was indicated by *, and *p* < 0.01 by **.

## 3 Results

### 3.1 Unsupervised machine learning clustering and differential analysis of wheezing disease recovery samples in infants and children

**Figure 1:**
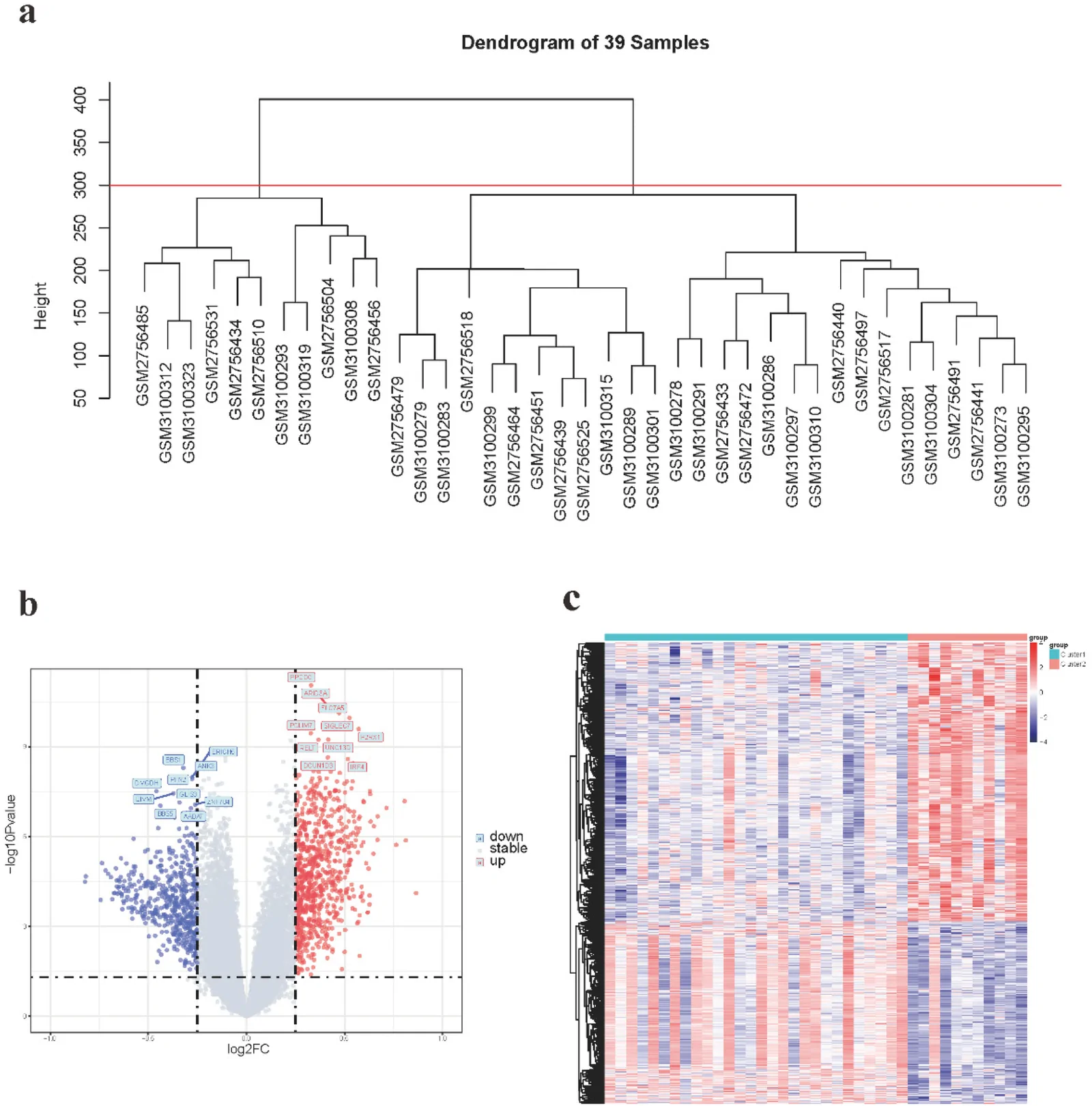
Unsupervised learning hierarchical clustering analysis **(a)** DEGs of two clusters **(b)** and differential gene heatmap **(c)**.

We integrated data from wheezing recovery samples of pediatric patients from GSE113209 and GSE103166. After performing principal component analysis (PCA) for dimensionality reduction, we used unsupervised machine learning hierarchical clustering analysis to identify two distinct clusters among the recovery samples (Figure 1a). Differential analysis between the two clusters (Cluster 2 vs. Cluster 1) revealed a total of 1,308 differentially expressed genes (DEGs; nominal *p* < 0.05, |log2FC| >0.25), with 791 upregulated and 517 downregulated genes (Figures 1b,c); 1,273 DEGs remained significant after Benjamini-Hochberg FDR correction. Age and sex were not significantly associated with cluster assignment (Supplementary File S1).

### 3.2 Functional enrichment analysis and immune infiltration analysis of subcluster DEGs

**Figure 2:**
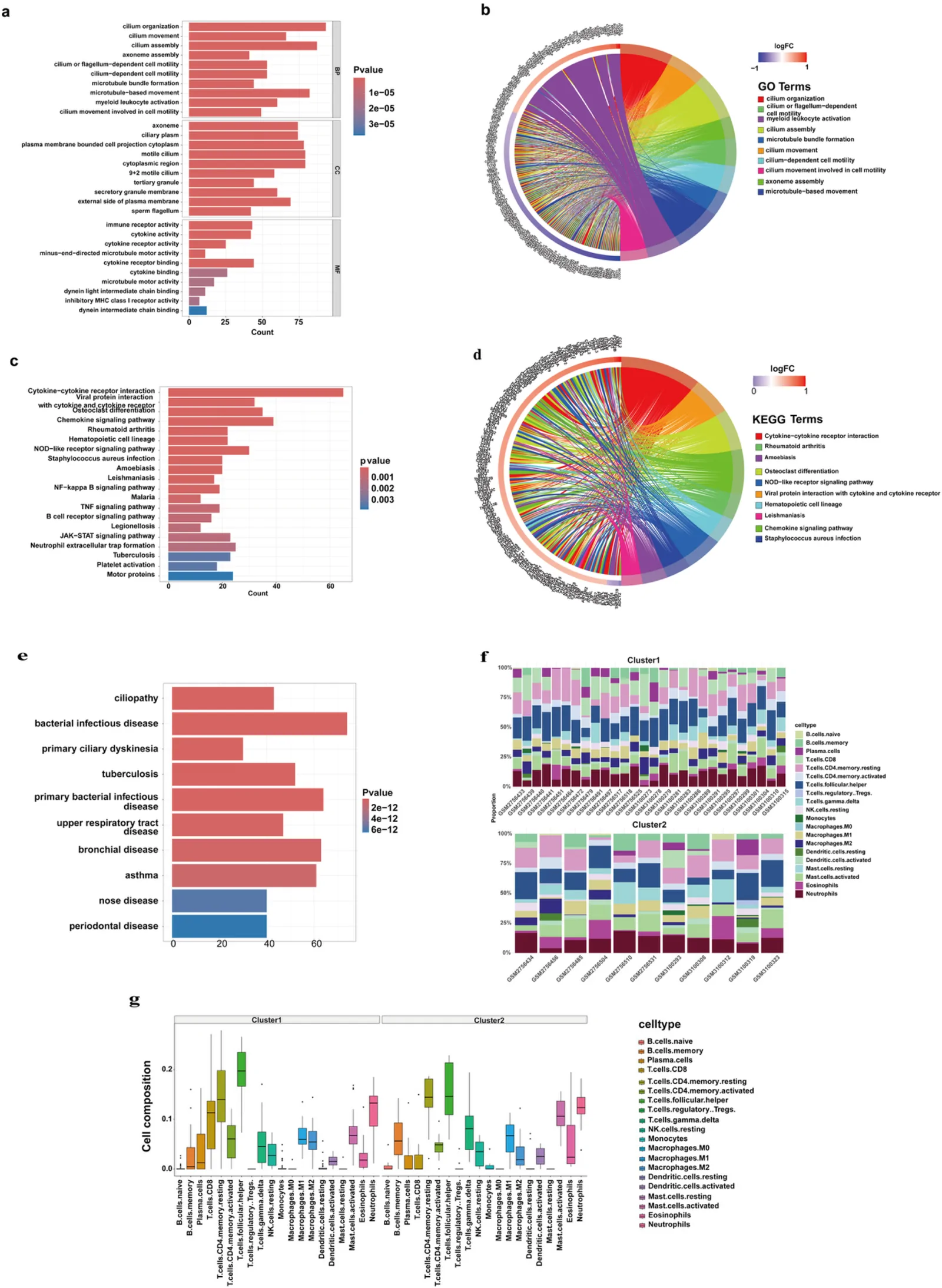
DEGs enrichment analysis and immune infiltration analysis. Classification results of GO analysis, MF: Molecular Function; BP: Biological Process; CC: Cellular Components **(a)** GO analyzes chord charts **(b)**. KEGG analysis results **(c)**. KEGG analyzes chord charts **(d)**. Results OF Disease Ontology analysis. The proportion stacking diagram of immune infiltration analysis, different colors represent different immune cells, Cluster1 on the top and cluster2 on the bottom **(f)**. Proportion box plot of immune infiltration analysis, different colors represent different immune cells, Cluster1 on the left and cluster2 on the right **(g)**.

We used the ClusterProfiler R package for GO and KEGG enrichment analysis. GO analysis revealed that DEGs were involved in processes including cilium movement, cilium organization, ciliary plasm, axoneme, cytokine activity, immune receptor activity, and inhibitory MHC class I receptor activity (Figure 2a). Upregulated DEGs were more strongly associated with myeloid leukocyte activation, while downregulated DEGs were more related to cilium assembly and movement (Figure 2b). KEGG analysis indicated that DEGs were predominantly involved in pathways such as cytokine-cytokine receptor interaction, viral protein interaction with cytokine and cytokine receptor, osteoclast differentiation, NOD-like receptor signaling pathway, NF-*κ*B signaling pathway, TNF signaling pathway, and JAK-STAT signaling pathway, with upregulated DEGs more represented in these pathways (Figures 2c,d). Disease Ontology (DO) analysis identified significant associations of DEGs with bacterial infectious diseases, primary ciliary dyskinesia, primary bacterial infectious diseases, bronchial diseases, and asthma (Figure 2e). Immune infiltration analysis using CIBERSORT revealed that, compared to Cluster 1, Cluster 2 samples exhibited increased infiltration of eosinophils, mast cells, and memory B cells, whereas plasma cells and CD8+ T cell infiltration were lower (Figures 2f,g).

### 3.3 Combined analysis of differentially expressed genes and persistent wheezing/asthma gene sets

**Figure 3:**
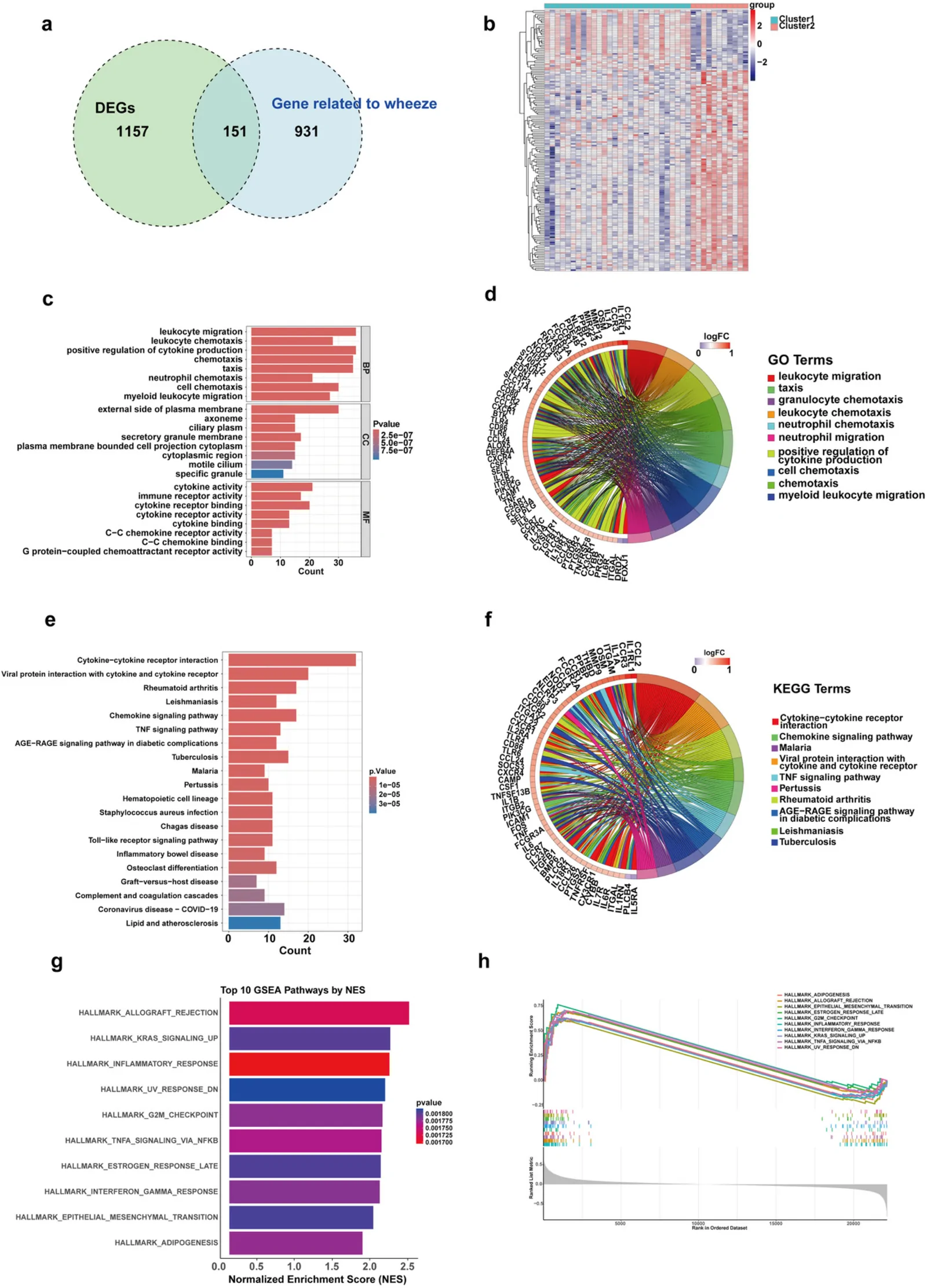
Enrichment analysis of genes related to persistent wheezing/asthma. Intersection of DEGs and genes associated with persistent wheezing disease **(a)** Heatmap of genes associated with wheezing **(b)** Classification results of GO analysis for genes associated with wheezing, MF: Molecular Function; BP: Biological Process; CC: Cellular Components **(c)** GO analyzes chord charts **(d)**. KEGG analysis results for genes associated with wheezing **(e)** KEGG analyzes chord charts **(f)**. Bar graph of the results of GSEA analysis of genes associated with wheezing **(g)**. Graph of the results of GSEA analysis of wheezing related genes **(h)**.

Based on the DEGs and enrichment analysis results, we hypothesized that DEGs are associated with elevated inflammation and reduced ciliary function. DO analysis results indicated a strong association of DEGs with bronchial diseases and asthma, and immune infiltration analysis revealed an increase in mast cells and eosinophils in Cluster 2. To further investigate the potential association of the two subclusters identified by hierarchical clustering with persistent wheezing and asthma progression, we intersected 1,082 persistent wheezing/asthma-related genes obtained from the Gencard database (Supplementary File S2) with our DEGs. This revealed 151 DEGs in the pediatric wheezing disease recovery samples that overlapped with persistent wheezing/asthma-associated gene sets, suggesting shared inflammatory pathways rather than direct asthma prediction (Figures 3a,b). Enrichment analysis of these 151 DEGs showed that GO terms primarily included leukocyte migration, external side of the plasma membrane, and cytokine activity (Figures 3c,d), while KEGG pathways included cytokine-cytokine receptor interaction, viral protein interaction with cytokine and cytokine receptor, and TNF signaling pathway (Figures 3e,f) (GO and KEGG enrichment analysis of gene correlation heat maps are provided in the Supplementary File S3, Supplementary Figures S1, S2). GSEA analysis of the 1,082 persistent wheezing/asthma-related genes in the pediatric wheezing disease recovery samples indicated upregulation of pathways such as allograft rejection, KRAS signaling, inflammatory response, and TNFA signaling via NF*κ*B (Figures 3g,h).

### 3.4 Identification of potential biomarkers for cluster 2 in the wheezing disease recovery cohort

**Figure 4:**
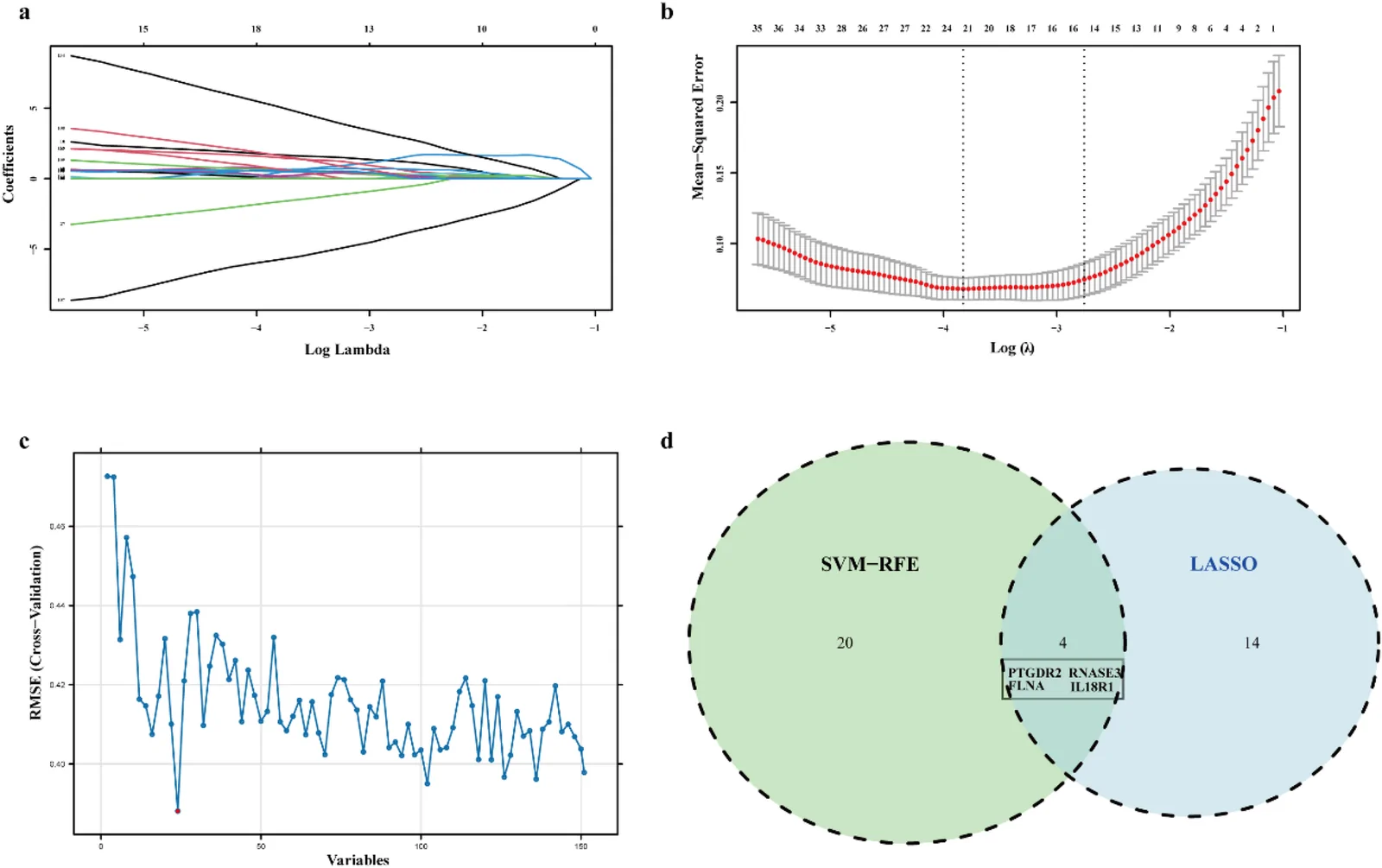
Selection of biomarker candidates for CLUSTER 2. Lasso regression feature selection curve **(a)** Tunning feature screening in the LASSO model **(b)** A plot of biological marker screening via the SVM-RFE arithmetic **(c)**. Venn graph displaying 4 biomarkers shared by LASSO and SVM-RFE **(d)**.

From the clustering analysis, differential gene enrichment analysis, and integration with the persistent wheezing/asthma gene set, we identified two subclusters (Cluster 1 and Cluster 2) in the pediatric wheezing disease recovery cohort. Cluster 2 was more indicative of potential disease progression towards persistent inflammation. To further investigate potential molecular markers indicating Cluster 2 within the recovery cohort, we applied lasso regression (Figures 4a,b) and SVM-RFE (Figure 4c) analyses to the 151 persistent wheezing/asthma-associated DEGs. Lasso regression identified 18 potential biomarkers, while SVM-RFE identified 24. To identify the most robust markers, we focused on the four candidates consistently selected by both complementary algorithms (FLNA, PTGDR2, RNASE3, and IL18R1), which were associated with a shift towards Cluster 2 in recovery samples (Figure 4d).

### 3.5 Expression and diagnostic performance of biomarkers

**Figure 5:**
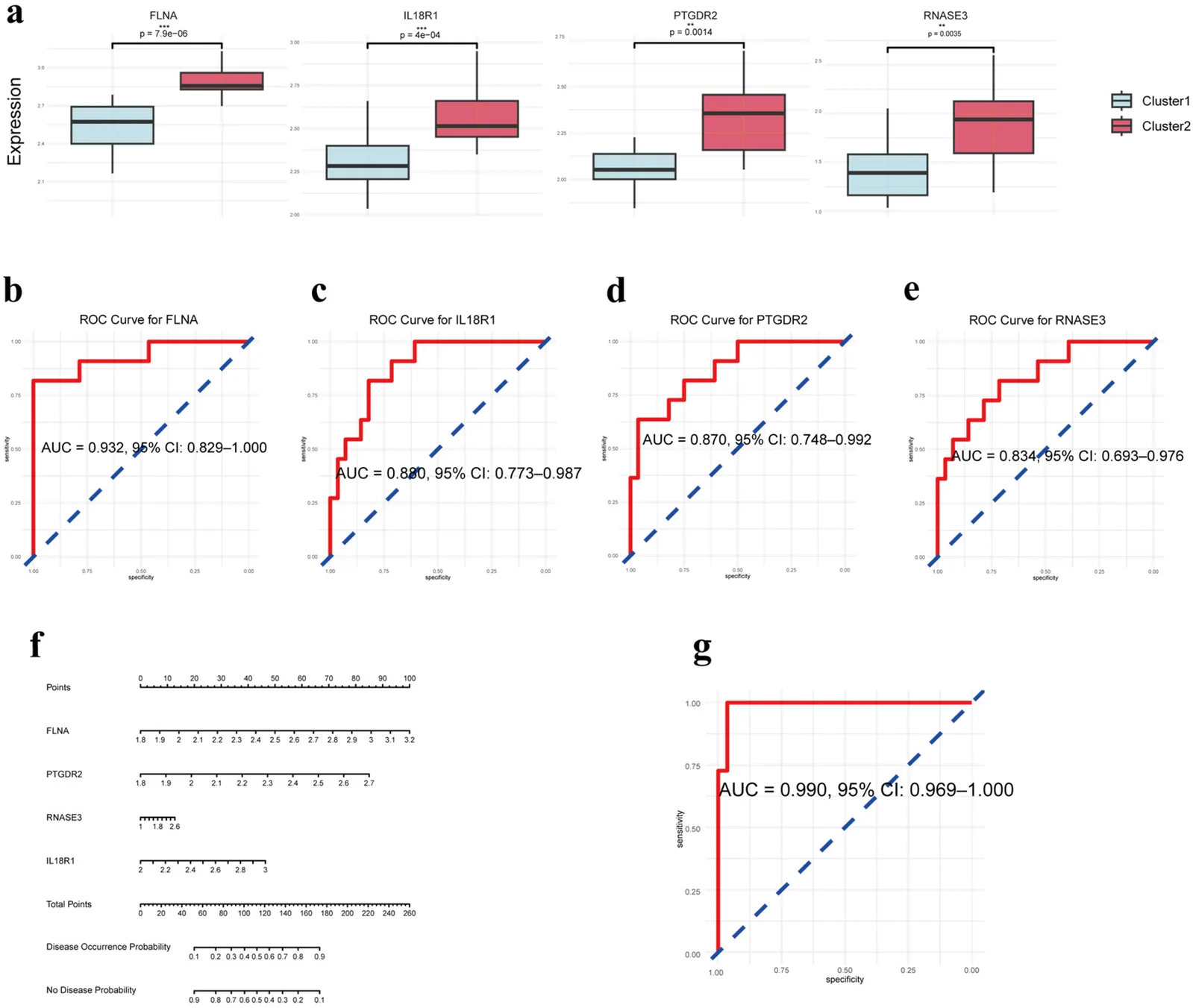
Biomarker expression and validation of subgroup identification in the wheezing recovery cohort. Expression levels of FLNA, PTGDR2, RNASE3, and IL18R1 in a convalescent cohort of children with wheezing **(a)** The ROC curves of FLNA **(b)**, PTGDR2 **(c)**, RNASE3 **(d)**, and IL18R1 **(e)** in identifying the two subgroups in the convalescent cohort of wheezing. AUC 95% confidence intervals were calculated using the DeLong method. Nomogram for combination of FLNA, PTGDR2, RNASE3, and IL18R1 to identify subgroups in cohort of recovery phase wheezing **(f)**. ROC curve of the nomogram model in the cohort **(g)** AUC: Area under the curve.

In the previous analysis, we found that FLNA, PTGDR2, RNASE3, and IL18R1 were indicative of Cluster 2 in the wheezing disease recovery samples. To validate the predictive performance of these genes, we first examined their expression levels in the wheezing disease recovery cohort (Figure 5a). Results showed that FLNA, PTGDR2, RNASE3, and IL18R1 expression levels were higher in Cluster 2 than in Cluster 1. Logistic regression analysis and ROC curve construction indicated that each gene could predict subgroup distribution within the wheezing disease recovery cohort (Figures 5b–e), with training AUC values of 0.932 (95% CI: 0.829–1.000) for FLNA, 0.870 (95% CI: 0.748–0.992) for PTGDR2, 0.834 (95% CI: 0.693–0.976) for RNASE3, and 0.880 (95% CI: 0.773–0.987) for IL18R1. To assess overfitting, leave-one-out cross-validation (LOOCV) was performed, yielding LOOCV-AUC values of 0.893 for FLNA (95% CI: 0.734–1.000), 0.854 for IL18R1 (95% CI: 0.733–0.975), 0.825 for PTGDR2 (95% CI: 0.668–0.981), and 0.763 for RNASE3 (95% CI: 0.569–0.957). The combined nomogram model achieved a LOOCV-AUC of 0.786 (95% CI: 0.549–1.000), which was lower than the apparent training AUC of 0.990, indicating some degree of overfitting expected given the small sample size (Supplementary File S5). Accordingly, we constructed a nomogram model based on the expression levels of FLNA, PTGDR2, RNASE3, and IL18R1 (Figure 5f). The ROC curve showed that the nomogram model accurately predicted subgroup distribution within the wheezing disease recovery cohort (Figure 5g).

### 3.6 Association of FLNA, PTGDR2, RNASE3, and IL18R1 with immune infiltration

**Figure 6:**
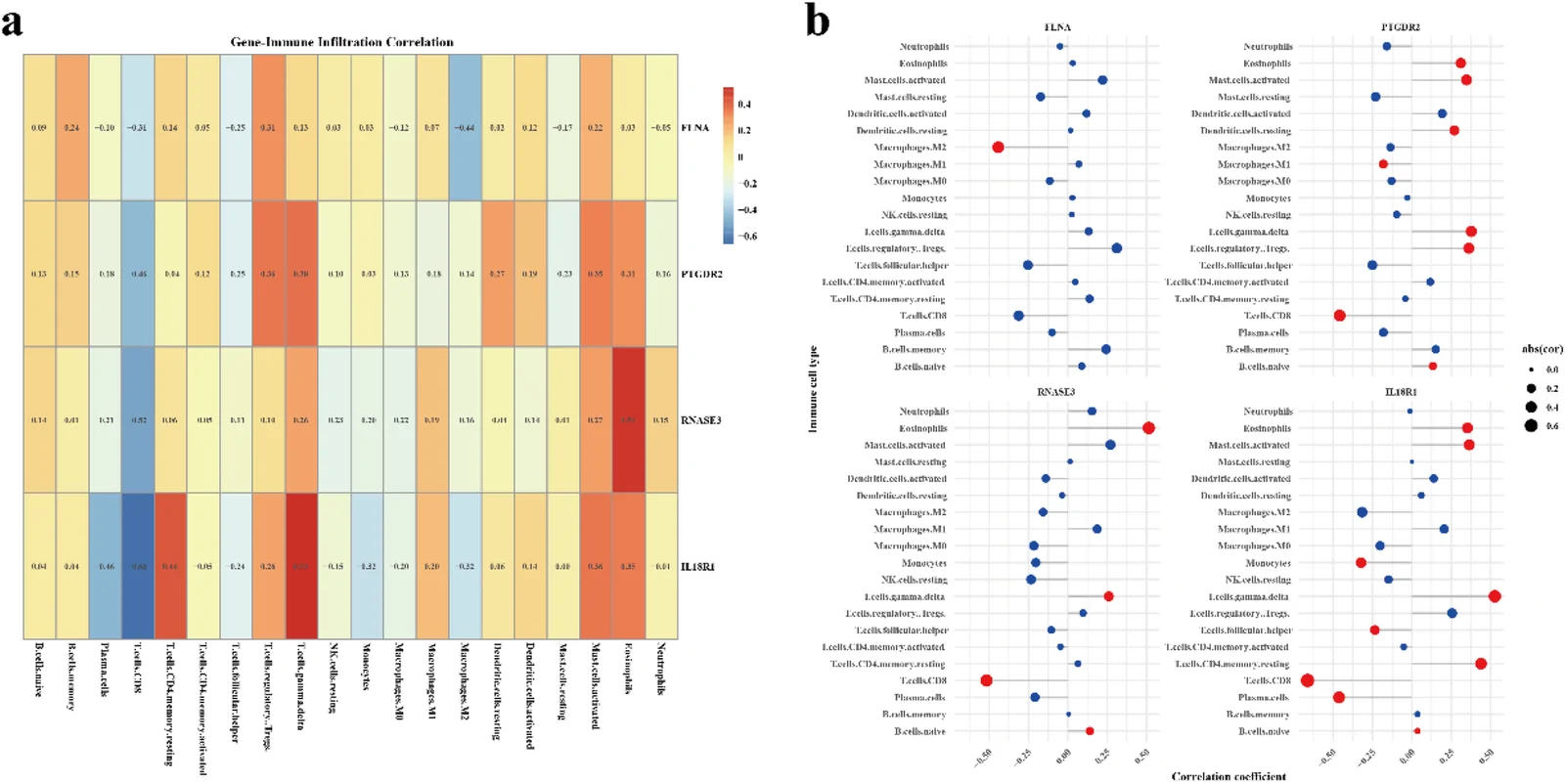
Correlation between biomarkers and infiltrating immune cells in 2 clusters of the wheeze recovery cohort. Heat map of correlation between biomarkers and immune infiltration **(a)** FLNA, PTGDR2, RNASE3, IL18R1 and infiltrating immune cells **(B)**.

Immune cell infiltration is closely related to the progression of airway inflammatory diseases. Thus, we analyzed the correlation between biomarkers FLNA, PTGDR2, RNASE3, and IL18R1 with immune infiltration across the two subclusters. In previous analyses, we observed increased infiltration of eosinophils, mast cells, and memory B cells and decreased plasma cells and CD8+ T cells in Cluster 2 compared to Cluster 1 (Figures 2f,g). The heatmap of correlations between FLNA, PTGDR2, RNASE3, IL18R1, and immune infiltration showed that each biomarker was associated with immune cell infiltration (Figure 6a). Further analysis revealed that higher expression of FLNA was negatively correlated with M2 macrophage polarization, while increased expression of PTGDR2, RNASE3, and IL18R1 was positively correlated with eosinophil and *γδ*T cell infiltration and negatively correlated with CD8+ T cell infiltration. Additionally, PTGDR2 and IL18R1 were associated with activated mast cell infiltration (Figure 6b). These findings suggest that FLNA, PTGDR2, RNASE3, and IL18R1 may modulate immune cell infiltration in upper respiratory tract specimens from recovering pediatric wheezing patients. Notably, the association between IL18R1 and immune infiltration was more prominent, prompting validation of the relationship between IL18R1 and wheezing episodes in an external pediatric cohort.

### 3.7 IL18R1 demonstrates strong predictive performance in the cohort of children with the first onset of wheezing

**Figure 7:**
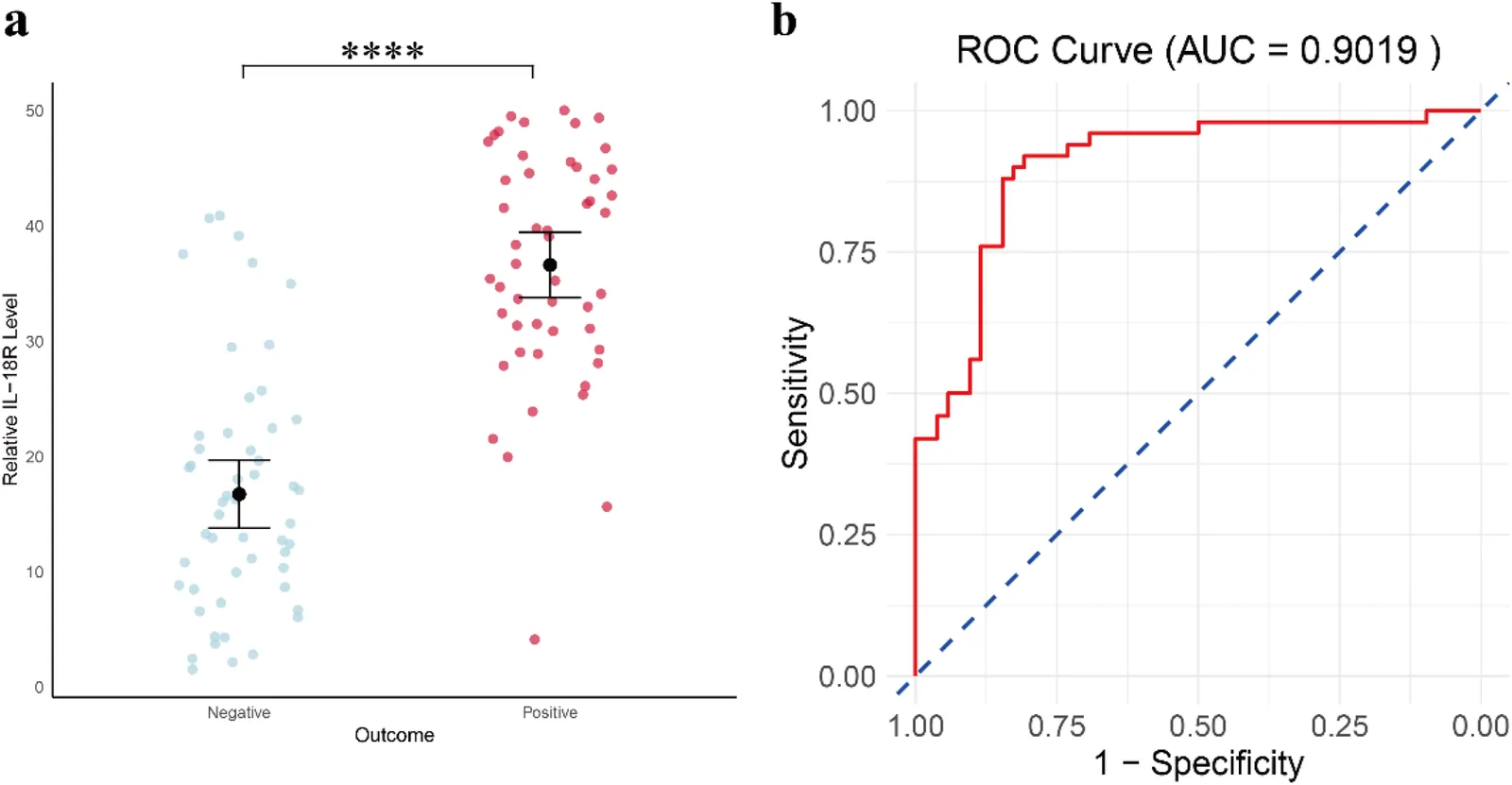
IL18R1 levels and predictive performance in the cohort of children with the first onset of wheezing. In cases with a positive outcome, nasopharyngeal swab samples showed a significant upregulation of IL18R1 levels **(a)** the predictive performance of IL18R1 in this cohort is shown by the ROC curve **(b)** AUC: Area Under the Curve.

To further validate the predictive efficacy of the biomarkers we identified, we recruited a cohort of children experiencing their first episode of wheezing and conducted a 3-month follow-up. Baseline characteristics of the cohort are presented in Table 2, with complete data provided in the Supplementary File S4. A positive outcome was defined as the recurrence of at least one episode of wheezing determined by physician diagnosis during the 3-month follow-up period. Among the 102 children with complete data, 50 exhibited a positive outcome. Gender and age showed no significant association with outcome (Age: Cohen's d = −0.006, 95% CI: −0.398 to 0.388, *p* = 0.978; Gender: OR = 1.605, 95% CI: 0.734–3.507, *p* = 0.322). The rate of viral infection positivity was also similar between groups (OR = 1.091, 95% CI: 0.500–2.381, *p* = 0.984). The rate of viral infection positivity was slightly higher in the positive outcome group compared to the negative outcome group, although no significant difference was observed. Among the four biomarkers identified by machine learning, only IL18R1 was assessed in this external cohort due to resource constraints. IL18R1 showed a significant upregulation in the positive outcome group (Figure 7a), and the ROC curve analysis further confirmed a significant association between IL18R1 and positive outcomes (Figure 7b).

**Table 2:** Baseline characteristics of the cohort of children with the first onset of wheezing.

		Non-recurrence Group	Recurrence Group	Effect Size (95% CI:)	*P* value
Number (*n*)		52	50		
Age (months)^a^		20.16 ± 8.49	20.11 ± 8.82	Cohen's d: **−**0.006 **(**−0.398 to 0.388**)**	0.978
Gender	Female (*n*)	23	28	OR:1.605 (0.734 to 3.507)	0.322
	Male (*n*)	29	22		
Relative IL18R1 level^a^		16.73 ± 10.56	36.63 ± 9.95	Cohen's d:1.937 (1.461to 2.414)	**<0**.**001**
Virus infection (%)		53.84%	56%	OR:1.091 (0.500 to 2.381)	0.984

a Values for Age and Relative IL18R1 level are presented as mean ± standard deviation. *P* values were calculated using Student's *t*-test for continuous variables and chi-square test for categorical variables. Effect sizes are reported as Cohen's d (with 95% CI) for continuous variables and odds ratios (OR, with 95% CI) for categorical variables.

Bold font indicates significant differences.

## 4 Discussion

Wheezing that occurs in infancy may be caused by viral infections, environmental factors, and genetic susceptibility (20). Although most children's wheezing symptoms subside with age, some cases may progress to persistent wheezing, often characterized by chronic airway inflammation, airway hyperreactivity, and genetic susceptibility, which can ultimately lead to a diagnosis of asthma. Therefore, early identification of children at risk for progression to persistent wheezing is crucial.

The recovery phase following a wheezing episode, which occurs after the acute phase, represents a transitional period in which symptoms may either improve or progress toward persistent wheezing/asthma. However, this phase is often overlooked once symptoms appear controlled, resulting in missed opportunities for intervention that could potentially alter the progression to persistent wheezing (21, 22). To address this, we collected upper respiratory tract RNA-seq data of pediatric wheezing recovery samples from the GEO database. Through unsupervised clustering analysis, we identified two distinct subgroups within the recovery phase samples. This observation aligns with findings in similar studies (14–16), which also reported the emergence of new subgroups in PCA clustering of upper respiratory samples from wheezing patients, different from their initial grouping. However, previous studies have not considered the potential implications of these subgroups for the prognosis of pediatric wheezing diseases.

We are particularly interested in determining whether these two clusters exhibit distinct clinical outcomes within the cohort, investigating their inherent characteristics and association with persistent wheezing—specifically, whether certain cases demonstrate heightened susceptibility to developing persistent wheezing or asthma. Unfortunately, due to a lack of clinical follow-up data, we instead conducted a summary analysis of clustering features based on RNA-seq data to provide insights into these patterns. This approach aims to elucidate potential molecular and immunological distinctions that could serve as early indicators of disease outcomes in pediatric wheezing recovery cases.

By integrating RNA-seq data from multiple GEO datasets, we identified two distinct clusters within the recovery phase samples, with Cluster 2 demonstrating characteristics suggestive of an increased risk for progression toward persistent wheezing or asthma. Through further analysis, we identified FLNA, PTGDR2, RNASE3, and IL18R1 as key biomarkers, showing significant associations with specific immune cell infiltrations. These findings provide valuable insights into the immune modulation and molecular mechanisms underlying the progression of pediatric wheezing disease and highlight potential targets for early intervention.

The identification of two clusters within the recovery phase samples, each exhibiting distinct immune infiltration profiles, suggests heterogeneity in immune responses during the post-wheezing recovery period. Cluster 2, characterized by increased infiltration of eosinophils, mast cells, and memory B cells, alongside decreased infiltration of plasma cells and CD8+ T cells, appears to represent a pro-inflammatory profile. This immune landscape aligns with findings in persistent asthma and suggests that certain immune cell types, such as eosinophils and mast cells, could play a role in the transition from transient wheezing to a more chronic, asthma-like phenotype (11, 18, 23).

Our focus on FLNA, PTGDR2, RNASE3, and IL18R1 as potential biomarkers stems from their distinct expression patterns between clusters and their significant associations with immune cell infiltration. Specifically, the positive correlation of PTGDR2, RNASE3, and IL18R1 with eosinophil and *γδ*T cell infiltration, suggests that these genes may influence immune cell recruitment and activation. For example, the association of PTGDR2 and IL18R1 with activated mast cell infiltration points to their possible involvement in pro-inflammatory signaling pathways that could exacerbate airway hyperresponsiveness and inflammation, conditions frequently observed in asthma (24).

Furthermore, pathway enrichment analysis of differentially expressed genes (DEGs) in Cluster 2 revealed upregulation in pathways involved in cytokine signaling, NF-kappa B activation, and JAK-STAT signaling, which are known to play critical roles in inflammatory responses (18, 25). The significant association of DEGs with diseases like bronchial disease and asthma further supports the hypothesis that Cluster 2 represents a subgroup of patients with a heightened risk of progressing to persistent wheezing or asthma.

Their associations with distinct immune cell types suggest that they may not only serve as markers but could also play functional roles in disease pathogenesis by modulating immune responses within the respiratory tract. Targeting these biomarkers could potentially offer new therapeutic approaches aimed at reducing inflammation and preventing the development of chronic airway diseases.

The identification of immune cell-specific correlations with these biomarkers may also inform future therapeutic strategies. For instance, if eosinophil and mast cell infiltration contribute to the transition from transient wheezing to asthma, therapies targeting these cells, or the pathways regulating their activity, might be beneficial. Such approaches could focus on reducing eosinophilic inflammation, stabilizing mast cells, or modulating T cell activity to prevent long-term airway remodeling and hyperresponsiveness (12, 26).

Despite the insights gained from this study, several limitations must be acknowledged. First, the discovery sample size is relatively small (*n* = 39), which may limit the generalizability of our findings and increase the risk of overfitting in machine learning models. This is reflected in the gap between the apparent training AUC of the combined nomogram model (0.990) and its LOOCV-AUC (0.786). Individual biomarkers showed more stable cross-validated performance, with FLNA achieving the highest LOOCV-AUC (0.893). The wide confidence intervals reflect the limited sample size and class imbalance (28 Cluster 1 vs. 11 Cluster 2); larger cohorts are needed to obtain more precise internal validation estimates. Second, the use of log-transformed TPM values rather than raw counts for differential expression analysis, while necessitated by GEO data availability, may introduce composition effects, particularly for immune-related genes that vary substantially in total RNA proportion across samples. Third, CIBERSORT was originally developed for bulk tissue transcriptomes; its performance on nasal swab samples, which contain heterogeneous cell types with different RNA yields, is not well validated, and results should be interpreted with caution. Fourth, the “recovery phase” timing relied on clinician judgment, which may introduce inter-clinician variability. Fifth, the external validation cohort was followed for only 3 months, which captures early recurrences but is insufficient to assess progression to persistent wheezing or asthma, which typically develops over years. Sixth, our external cohort exclusively comprised individuals of East Asian descent, which differs ethnically from the training cohort and limits cross-population generalizability. Seventh, the inclusion of GSE103166 (children with pre-existing asthma diagnoses) in the discovery cohort may have influenced the clustering results; the identified pro-inflammatory Cluster 2 may partially reflect underlying asthma biology rather than *de novo* risk stratification. Eighth, the 3-month follow-up duration, chosen pragmatically based on clinical practice patterns, is insufficient to assess progression to persistent wheezing or asthma, which typically develops over years; the study should be viewed as providing early evidence of short-term wheezing recurrence rather than long-term asthma prediction. Ninth, we did not systematically collect clinical severity indicators (e.g., respiratory support requirements, length of hospital stay) for the external cohort; the association between IL18R1 levels and disease severity warrants further investigation. Tenth, transcriptomic data were not collected for the external cohort, so the original clustering structure could not be independently reproduced; the external cohort analysis therefore validates the clinical relevance of IL18R1 as a biomarker, but not the clustering-based subgroup classification itself. Eleventh, comprehensive data on established confounders such as prematurity, personal history of atopy, and family history of allergic disease were not systematically collected for the external cohort. These are well-established risk factors for wheezing persistence and asthma development and their absence limits the ability to adjust for potential confounding in the association between IL18R1 and wheezing recurrence. Most importantly, our study merely identified a subgroup within the wheezing recovery phase cohort exhibiting pro-inflammatory tendencies. We explicitly emphasize that these findings should not be construed as identifying potential asthma cases. Rather, we propose that this particular subgroup warrants clinical attention. Whether this population will ultimately progress to asthma remains uncertain and requires validation through longitudinal follow-up studies with larger sample sizes.

Future research should focus on validating these biomarkers in larger, independent cohorts to confirm their utility in predicting disease progression in pediatric wheezing recovery cases. Moreover, mechanistic studies are needed to understand the exact roles of IL18R1 in modulating immune responses. These studies could investigate how these genes interact with cytokine signaling, inflammatory pathways, and immune cell recruitment, which are critical processes in the pathophysiology of asthma and other chronic respiratory diseases.

Additionally, integrating the biomarker with clinical data to develop a comprehensive predictive model could improve the accuracy of risk stratification for persistent wheezing or asthma. Such a model could combine immune cell profiles, gene expression data, and clinical indicators to provide a more holistic approach to patient management. Lastly, exploring therapeutic strategies that target the pathways associated with these biomarkers could offer new avenues for early intervention in high-risk pediatric populations, potentially altering the disease course and improving long-term respiratory health outcomes.

## 5 Conclusion

This study underscores the heterogeneity of immune responses during the recovery phase of pediatric wheezing disease. Notably, Cluster 2 appears to exhibit a pro-inflammatory tendency, and its gene expression profile and immune infiltration characteristics partially overlap with those observed in persistent wheezing/asthma. IL18R1 emerges as a potential biomarker for Cluster 2, supported by our external cohort findings that children experiencing wheezing recurrence displayed significantly higher IL18R1 levels during the recovery phase of their initial wheezing episode.

In summary, our study demonstrates the necessity of identifying heterogeneity within wheezing recovery-phase samples, with IL18R1 serving as a potential biomarker for those with pro-inflammatory tendencies. We emphasize that the pro-inflammatory subpopulation within wheezing recovery samples warrants increased clinical attention and propose that higher-level research institutions should further investigate this subgroup to improve predictive models for identifying individuals at risk of asthma development.

## Data Availability

Publicly available datasets were analyzed in this study. This data can be found here: GSE113209 and GSE103166 in Gene Expression Omnibus (GEO).
