# Supplementary material for: Machine learning reveals immune heterogeneity in recovery-phase samples of pediatric wheezing
Source: Front Pediatr. 2026 Aug 24;14:1878743. doi: 10.3389/fped.2026.1878743 (PMC13547287; doi:10.3389/fped.2026.1878743)
Supplement: Supplementary file 2 [file Datasheet3.docx]

**Figure S1 heat map of wheezing related gene in DEGs and GO terms**

**Figure S2 heat map of wheezing related gene in DEGs and KEGG pathway**
